# Chemical-induced disease extraction via recurrent piecewise convolutional neural networks

**DOI:** 10.1186/s12911-018-0629-3

**Published:** 2018-07-23

**Authors:** Haodi Li, Ming Yang, Qingcai Chen, Buzhou Tang, Xiaolong Wang, Jun Yan

**Affiliations:** 1 Key Laboratory of Network Oriented Intelligent Computation, Harbin Institute of Technology, Shenzhen, Guangdong China; 2 Shenzhen Calligraphy Digital Simulation Technology Engineering Laboratory, Harbin Institute of Technology, Shenzhen, Guangdong China; 3 Pharmacy Department, Shenzhen Second People’s Hospital, First Affiliated Hospital of Shenzhen University, Guandong, Shenzhen, China; 4 Yidu Cloud (Beijing) Technology Co., Ltd, Beijing, China

**Keywords:** Chemical-induced disease, Relation extraction, Deep learning, Convolutional neural network

## Abstract

**Background:**

Extracting relationships between chemicals and diseases from unstructured literature have attracted plenty of attention since the relationships are very useful for a large number of biomedical applications such as drug repositioning and pharmacovigilance. A number of machine learning methods have been proposed for chemical-induced disease (CID) extraction due to some publicly available annotated corpora. Most of them suffer from time-consuming feature engineering except deep learning methods. In this paper, we propose a novel document-level deep learning method, called recurrent piecewise convolutional neural networks (RPCNN), for CID extraction.

**Results:**

Experimental results on a benchmark dataset, the CDR (Chemical-induced Disease Relation) dataset of the BioCreative V challenge for CID extraction show that the highest precision, recall and F-score of our RPCNN-based CID extraction system are 65.24, 77.21 and 70.77%, which is competitive with other state-of-the-art systems.

**Conclusions:**

A novel deep learning method is proposed for document-level CID extraction, where domain knowledge, piecewise strategy, attention mechanism, and multi-instance learning are combined together. The effectiveness of the method is proved by experiments conducted on a benchmark dataset.

## Background

Nowdays, there is more and more literature published with rich domain knowledge. The first step to reuse literature is to extract biomedical information from literature. Chemical-induced disease (CID), which refers to adverse drug reactions, is a type of important information, which can be used for drug safety monitoring and medicine development [1], has attracted more and more attentions.

During the last decade, there have been a large number of methods proposed for CID extraction [2], which can be classified into three categories: 1) statistics-based methods, 2) rule-based methods, and 3) machine learning-based methods. The statistics-based methods determine CIDs according to the distributions of chemicals and diseases. For example, Chen et al. [3] discovered drug side effects by analyzing co-occurrences of drugs and adverse reactions in biomedical literature. Mao et al. [4] used a similar method to mine drug side effects from social media. The limitation of statistic-based methods lies in their low precision, although they usually achieves high recall. Khoo et al. [5] used manually-constructed graphical patterns derived from syntactic parse trees to extract causal relations between drugs and adverse events in MEDLINE abstracts. The rule-based methods usually need domain experts, constructing rules is time-consuming, and the manually-crafted rules are not easily applicable to other corpora. To increase generalizability of rules, Xu and Wang [6] provided a method to learn syntactic patterns from sentences containing known drug side effect pairs for drug side effect extraction from biomedical literature. The manchine learning-based methods are deployed for CID extaction due to some manually-annotated corpora, such as the corpus of the BioCreative V chemical-indcued disease relation (CDR) challenge [7] for CID extraction, are publically available. Support vector machine (SVM) is the most commonly used machine learning method. Xu et al. [8] won the BioCreative V CDR challenge using an SVM-based system. The feature engineering of the SVM-based system is terrible. To avoid fussy feature engineering, deep learning methods were applied to CID extraction [9], including convolutional neural networks (CNN) [10] and long short term memory neural networks (LSTM) [11]. In these systems, domain knowledge about adverse drug reactions, and some new techniques, such as piecewise strategy [12] and attention mechanism [13], widely used in other domains are not considered. Subsequently, Li et al. [14] adopted piecewise CNN to extract chemical-disease relations contained in intra-sentence and inter-sentence using a uniform model. Gu [15] improved the CNN model by adding syntactic information of cross-sentence, and the performance has been further improved. However, all these methods extract chemical-disease relations from single sentences or adjacent sentences. None of them consider document-level information. In a document, two entities usually do not appear only once, and it is difficult to determine which sentence or paragraph describes a relation or not. To facilitate efficient document-level relation extraction from biological text, Patrick [16] proposed Bi-affine Relation Attention Networks (BRAN), a combination of network architecture, multi-instance and multi-task learning. In this paper, we propose a novel document-level deep learning method for CID extraction, called recurrent piecewise convolutional neural networks (RPCNN). It should be noted that this paper is an extension of our previous paper [14].

## Methods

### Overview

There are usually two steps in chemical-induced disease extraction: 1) candidate generation – generating all possible related pairs of chemicals and diseases, denoted by <chemical, disease>; 2) candidate classification – determining whether each <chemical, disease> pair generated in the previous step is related.

### Candidate generation

Given a biomedical record with *m* chemical mentions and *n* disease mentions, all *m* × *n* < chemical, disease> pairs can be recognized as candidates. In this study, we combine <chemical, disease> pairs that have the same chemical and disease identifiers together to form a candidate, denoted by <chemical identifier, disease identifier>. An example of candidate generation is shown in Table 1, where given a record with 2 chemical mentions (i.e., “terbutaline”×2) and 4 disease mentions (i.e., “Cardiovascular complications”, “cardiovascular complications”, “andpreterm labor”×2), as the two chemical mentions has the same MeSH (Medical Subject Headings) [17] identifier (i.e., D013726) and 4 disease mentions correspond to 2 MeSH identifiers (i.e, cardiovascular complications – D002318 and preterm labor – D007752), two candidates, that is, <D013726, D002318 > and < D013726, D007752>, are generated. Each candidate is a document-level candidate corresponding with multiple < chemical, disease> pairs, and each <chemical, disease> pair is an instance. Therefore, there are eight instances corresponding to two candidates in Table 1.

**Table 1:** An example of candidate generation (Literature with chemical and disease mentions and their identifiers)

Position			Mention	Label		Identifier (MeSH)
start	end					
0	28		Cardiovascular complications	Disease		D002318
45	56		terbutaline	Chemical		D013726
71	84		preterm labor	Disease		D007752
93	121		cardiovascular complications	Disease		D002318
169	180		terbutaline	Chemical		D013726
185	198		preterm labor	Disease		D007752
Identifier (MeSH)	Chemical mention			Disease mention		
	position		mention	Position		mention
	start	end		start	end	
<D013726, D002318>	45	56	terbutaline	0	28	Cardiovascular complications
	45	56	terbutaline	93	121	Cardiovascular complications
	169	180	terbutaline	0	28	Cardiovascular complications
	169	180	terbutaline	93	121	Cardiovascular complications
	position			position		mention
	start	end		Start	End	
<D013726, D007752>	45	56		71	84	preterm labor
	45	56		185	198	preterm labor
	169	180		71	84	preterm labor
	169	180		185	198	preterm labor

Cardiovascular complications associated with terbutaline treatment for preterm labor

Abstract: Severe cardiovascular complications occurred in eight of 160 patients treated with terbutaline for preterm labor. Associated corticosteroid therapy and twin gestations appear to be predisposing factors. Potential mechanisms of the pathophysiology are briefly discussed

### Candidate classification

**Fig. 1:**
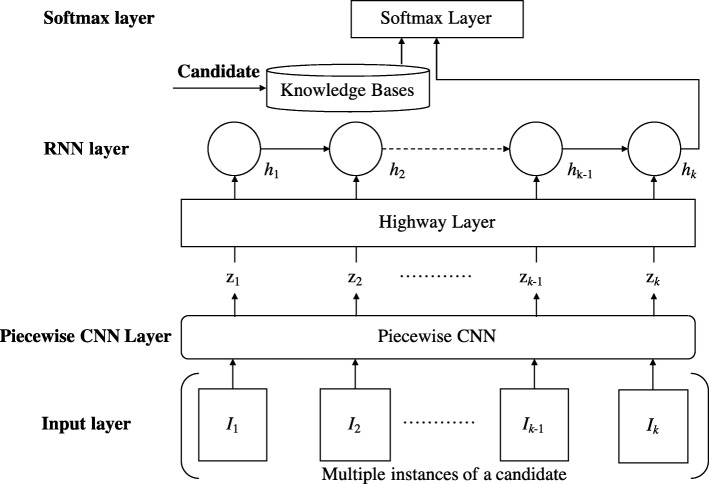
Architecture of recurrent piecewise convolutional neural networks (RPCNN) for multi-instance learning

A four-layer recurrent piecewise convolutional neural networks (RPCNN) is proposed for CID extraction as shown in Fig. 1, where piecewise CNN (the same as Li et al. [14]) is used to represent each instance of a candidate, and RNN is used to combine representations of each candidate’s instances in a record together to obtain the document-level representation of the candidate.

### Input layer

Given a candidate, the corresponding multiple instances *I*_0_, *I*_1_, …, *I*_*m*_ are arranged in descending order according to the length of context between the two entity mentions, which is measured by the number of words within the context. For each instance, we select the two entity mentions with context between them and context before or after them in the same sentence as the instance’s input. To distinguish chemical entity mentions and disease mentions, “*<ENTC > ...</ENTC>*” and “*<ENTD> ... </ENTD>*”, are further used to enclose them respectively. Then, an instance’s input is divided into three parts: 1) ***S***_**− 1**_: context before the first entity mention (e.g., “Severe ... with” before “*<ENTC>* terbutaline *</ENTC>*” in Table 2); 2) ***S***_**0**_: context between the two entity mentions (e.g., “for” in Table 2); and 3) ***S***_**1**_: context after the second entity mention (e.g., “.” after “*<ENTD>* preterm labor *</ENTD>*” in Table 2). Each word of an instance’s input is represented by word embedding and embeddings of positions relative to chemcial and disease mentions (see Table 2). For convenience, the lengths of all instances’ inputs (i.e., numbers of words within inputs) are set to the maximum (denoted by *l*). For instances with short input, paddings are appended to their input to make up the difference. Given an instance <*c*, *a* > with input *S* = *w*_1_*w*_2_…*w*_*l*_, suppose that the positions of *c* and *a* in *S* are *p*_*c*_ and *p*_*a*_ respectively, word *w*_*i*_ can be represented by ${x}_i={\left[{e}_{w_i}^{\mathrm{T}},{e}_{d_{ic}}^{\mathrm{T}},{e}_{d_{ia}}^{\mathrm{T}}\right]}^{\mathrm{T}}$, where ${e}_{w_i\in \mid V\mid }$, ${e}_{d_{ic}}$ and ${e}_{d_{ia}}$correspond to a *d*_*w*_-dimensional word embedding, a ${d}_{p^c}$-dimensional position embedding and a ${d}_{p^a}$-dimensional position embedding, *d*_*ic*_ = *i* − *p*_*c*_ and *d*_*ia*_ = *i* − *p*_*a*_ are relative distances from *w* to *c* and *a* respectively (−*n* + 1 ≤ *d*_*ic*_, *d*_*ia*_ ≤ *n* − 1), and ∣*V*∣ is the word vocabulary. Then *S* = *w*_1_*w*_2_*w*_3_…*w*_*l*_ is represented by a matrix$x=\left[{x}_1,{x}_2,\dots, {x}_l\right]\in {R}^{\left({d}_w+{d}_{p^c}+{d}_{p^a}\right)\times l}$.

**Table 2:**
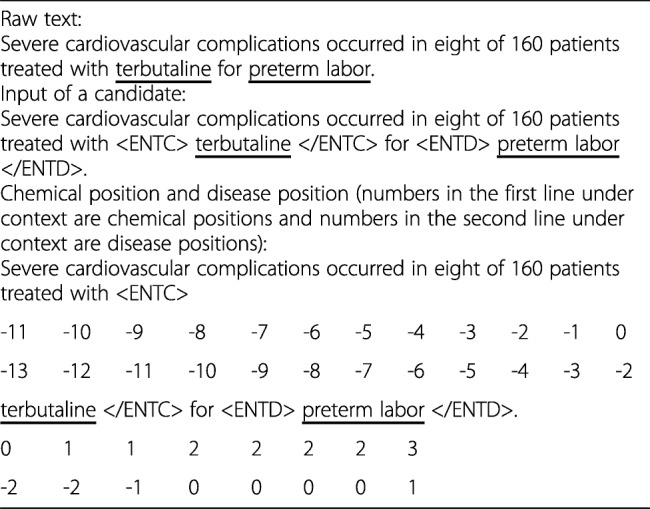
Example of chemical position and disease position

### Piecewise convolutional layer

The convolutional layer takes the matrix of each instance’ input *x*, and generates high-level feature vectors by convolving filters at multiple scales across *x*, where the filtes need to be learnt. Given a filter of size *k*, $t\in {R}^{\left({d}_w+{d}_{p^c}+{d}_{p^a}\right)\times k}$, for example, feature vector *f* = [*f*_1_, *f*_2_, …, *f*_*l* − *k* + 1_]^T^ ∈ *R*^*l* − *k* + 1^ is generated by sliding filter *t* across *S*’s input *x* with a convolution operator (take the rectified linear unit function (*Relu*) for example) as follows:

$$ {f}_i= Relu\left(t\bullet {x}_{i:i+k-1}+b\right), $$

where *x*_*i* : *i* + *k* − 1_ = [*x*_*i*_, *x*_*i* + 1_, …, *x*_*i* + *k* − 1_]^T^ is the context representation of *w*_*i*_*w*_*i* + 1_…*w*_*i* + *k* − 1_ within a *k*-word window, and *b* ∈ *R* is a bias. Each filter corresponds to a high-level feature vector. Therefore, how many filters determines how many feature vectors we can obtain.

To reduce the spatial size of the representation of each instance, the number of parameters and computation, max pooling is adopted to select some important features from all the features generated in the convolutional layer:

$$ \overline{f_t}=\max \left\{{f}_{t,1},\kern0.5em {f}_{t,2},\dots, {f}_{t,l+k-1}\right\}, $$

where (*f*_*t*, 1_,  *f*_*t*, 2_, …, *f*_*t*, *l* + *k* − 1)_ is the feature vector corresponding to filter *t*, and $\overline{f_t}$ is the maximum feature. If there are *q* filters, we a new *q*- dimensional vector is generated to represent *S*, denoted by$z={\left[\overline{f_1},\overline{f_2},\dots, \overline{f_q}\right]}^{\mathrm{T}}$. In addition, piecewise strategy that applies pooling to individual parts (i.e., *S*_−1_, *S*_0_ and *S*_1_), and concatenates the outputs of all pooling layers is also adopted in our study.

Before pooling, attention mechanism is used to measure feature importances for each class as follows:

$$ {\mathrm{G}}_t={f_t}^T\mathbf{M}\ {\boldsymbol{W}}^{\boldsymbol{classes}}, $$

$$ {A}_{i,j}=\frac{\exp \left({G}_{i,j}\right)}{\sum_{k=1}^n\exp \left({G}_{k,j}\right)}, $$

where *G* is a correlation matrix between features *f* for each filter *t* and relation class embedding ***W***^***classes***^, **M** and ***W***^***classes***^ are weight matrix need to be learnt, *A* is an attention matrix, *A*_*i*, *j*_ and *G*_*i*, *j*_ are the (*i*, *j*)-th entry of *A* and *G*, respectively. We use a uniform distribution to initialize **M**, and an identity matrix to initialize ***W***^***classes***^.

When the attention mechanism is adopted, the output of the pooling layer becomes:

$$ \overline{{f_{t,i}}^A}={\mathit{\max}}_j{\left({f}_tA\right)}_{i,j}, $$

where $\overline{{f_{t,i}}^A}$ and (*f*_*t*_*A*)_*i*, *j*_ are the *i*-th item of $\overline{{f_t}^A}$ and the (*i, j*)-th item of *f*_*t*_*A*, respectively.

### RNN layer

In this layer, RNN is used to model multiple instances of a candidate. For each instance *I*_*i*_, the corresponding RNN cell takes the output of the piecewise convolutional layer (i.e., *z*_***i***_) and the previously hidden vector *h*_*i* − 1_ as input, and output hidden vector *h*_*i*_ using a non-linear transformation function *ρ*, that is, *h*_*i*_ = *ρ*(*z*_***i***_, *h*_*i* − 1_). The last hidden vector *h*_*m*_ is used as the representation of multiple instances of a candidate, which is a document-level representation.

### Softmax layer

In this layer, a fully connected neural network is used for classification. The neural network takes the following two parts as input: 1) *h*_*m*_ from the RNN layer presented above; 2) features extracted from four domain knowledge bases, the same as Xu et al.’s system [8], as follows:

1. The CTD repository [18] that contains relationships between drugs and diseases, such as *inferred-association*, *therapeutic*, *marker/mechanism*, etc., manually summarized by experts.
2. The Drugs and Indications Database (MEDI) [19] that records common drugs with common indications.
3. SIDER (Drug Side Effects Database) [20] that records common drugs with common side effects.
4. Medical Subject Headings (MeSH) that records superordinate and inferior structural relationships between drugs and the diseases.

The one-hot features extracted from domain knowledges are first converted into dense features (denoted by *v*) by a 1-layer neural network. For candidate classification, we use the sigmoid function as follows:

$$ O\left({\boldsymbol{v}}^{\prime}\right)={\left(1+{e}^{\boldsymbol{u}\bullet {\boldsymbol{v}}^{\prime }}\right)}^{-1}, $$

where ${\boldsymbol{v}}^{\prime }={\left[{\boldsymbol{h}}_{\boldsymbol{m}}^{\mathbf{T}},{\boldsymbol{v}}^{\mathrm{T}}\right]}^{\mathrm{T}}$, and *u* is a weight vector.

### Dataset

Our method is evaluated on the CDR corpus of the BioCreative V challenge. This corpus contains 1500 manually annotated PubMed record, 1000 out of 1500 records are used as training and development sets, and the remainder 500 records as test set. In the training and development sets, there are 10,550 chemical mentions, 8426 disease mentions, corresponding to 3829 and 2973 MeSH identifiers respectively. and 2050 relations. In the test set, there are 5385 chemical mentions, 4424 disease mentions, corresponding to 1988 and 1435 MeSH identifiers respectively, and 1066 relations.

### Experimental settings

We start with a simple CNN-based system which only selects the last instance of every candidate in the input layer and does not use any one of domain knowledge, piecewise strategy or attention mechanism as baseline, and then compares it with CNN-based systems gradually using them and RPCNN. In addition, our best CNN-based and RPCNN-based systems are also compared with other state-of-the-art systems using a single machine learning method. Precision (P), recall (R) and F-score (F) are used to measure performance of all systems, which are calculated by the official evaluation tool of the BioCreative V organizer.

10-fold cross-validation is used to optimize all hyperparameters of our system on the training and development sets. Finally, *d*_*w*_, ${d}_{p^c}$ and ${d}_{p^a}$ are set to 30, 5 and 5 respectively. CBOW is deployed to initialize word embeddings on a large-scale unannotated corpus from Medline, and position embeddings are initialized by a uniform distribution. Filters at scales of 3 and 4 are selected and the numbers of filters are both set to 150. In the RNN layer, we used LSTM cell with 150 hidden states as the RNN cell. In the softmax layer, we follow Srivastava ‘s work [21] to randomly drop out units from networks to prevent overfitting during training, and set the dropout probability to 0.25. The number of units of the neural network for knowledge feature conversion is set to 120.

## Results

The precision, recall and F-score of the baseline system (CNN in Table 3, where the best performance in each column is in bold) are 50.47, 55.61 and 52.92%. Similar with [8], the CNN-based systems is significantly improved by the domain knowledge. Take the baselien system as an example, when the domain knowledge is added, the system’s F-score is improved by 15.72% (52.92% vs 68.64%). Both the piecewise strategy and attention mechanism are beneficial to the CNN-based systems and they are complementary to each other. For example, when the piecewise strategy is added into the baseline system (***CNN + piecewise*** in Table 3), the system’s F-score increases from 52.92 to 54.20%, while when the attention mechanism is added to the baseline system before pooling (***CNN + attention***), the F-score slightly increases from 52.92 to 52.99%. When both the piecewise strategy and attention mechanism are together added to the baseline system (***CNN + attention + piecewise***), the system’s F-score is further improved to 55.94%. When the domain knowledge is added, the effects of piecewise strategy and attention mechanism decrease. For example, the F-score difference between CNN using domain knowledge and ***CNN + piecewise*** using domain knowledge is 0.39%, while the F-score difference between corresponding systems without using domain knowledge is 1.28%. Among all CNN-based systems, the system that using domain knowledge, piecewise strategy and attention mechanism achieves highest F-score, which is 69.09%. The RPCNN-based system (***RPCNN***) outperforms ***CNN + attention + piecewise***. ***RPCNN*** without using domain knowledge achieves an F-score of 59.10%, higher than ***CNN + attention + piecewise*** by 3.16%, while ***RPCNN*** using domain knowledge achieves an F-score of 70.77%, which is higher than that of ***CNN + attention + piecewise*** by 1.68%.

**Table 3:** performance of our cnn-based and rpcnn-based systems for chemical-induced disease extraction

Methods	Without domain knowledge (%)			With domain knowledge (%)		
	*P*	*R*	*F*	*P*	*R*	*F*
CNN	50.47	55.61	52.92	63.70	74.40	68.64
CNN + piecewise	54.48	53.91	54.20	63.83	75.16	69.03
CNN + attention	48.40	58.54	52.99	62.28	76.58	68.69
CNN + attention+piecewise	57.80	54.20	55.94	59.97	81.49	69.09
RPCNN	55.17	63.63	59.10	65.24	77.21	70.77

Moreover, our best CNN-based and RPCNN-based systems are also compared with other state-of-art systems using a single machine learning method, including Xu et al.’s system developed for the CDR task of the BioCreative V challenge [8], Zhou et al.’s LSTM-based and CNN-based systems [9], Gu et al.’s CNN-based system [15] and Patrick et al.’s BRAN-based system. Table 4 list the results of comparison, where “/” denotes no result report, and the best performance in each column is in bold. Compared with Xu et al.’s system, our RPCNN-based system achieves much higher F-score no matter whether the domain knowledge is used. The difference between the systems without using domain knowledge is 5.21% (55.94% vs 50.73%), while that between the systems using domain knowledge is 3.61% (70.77% vs 67.16%). Compared with Zhou et al.’s systems, our RPCNN-based system also achieves much higher F-score. The F-score difference between our RPCNN-based system and Zhou’s systems arranges from 8.78 to 2.84%. Compared with Gu et al.’s system, though our CNN-based system does not perform better, our RPCNN-based system performs better by 1.90% in F-score. The Patrick et al.’s BRAN-based system achieves a higher F-score than our system by 3.00%, when it takes entity recogniton into account, which significantly improves the peformance of relation extraction. Without entity recognition multi-task objective, the BRAN-based’s F-score is only 55.50%.

**Table 4:** Comparison between our systems and other state-of-the-art systems

Methods	Without domain knowledge (%)			With domain knowledge (%)		
	*P*	*R*	*F*	*P*	*R*	*F*
Xu et al. [8]	59.60	44.00	50.73	65.80	68.57	67.16
Zhou et al. (LSTM) [9]	54.91	51.41	53.10	/	/	/
Zhou et al. (CNN) [9]	41.13	55.25	47.16	/	/	/
Gu et al. (CNN) [15]	59.70	55.00	57.20	/	/	/
Patrick et al. (BRAN) [16]	55.60	70.80	62.10	/	/	/
Our CNN	57.80	54.20	55.94	59.97	81.49	69.09
Our RPCNN	55.17	63.63	59.10	65.24	77.21	70.77

## Discussion

In this paper, we propose RPCNN for CID extraction, where domain knowledge, piecewise strategy, attention mechanism and multi-instance learning are naturally combined. The RPCNN-based system on a benchmark corpus shows state-of-the-art performance.

Similar to previous studies on CNN-based relation extraction in other domains, the piecewise strategy and attention mechanism are effective in our CNN-based system. In our system, the attention mechanism makes it have the ability to handle some cases when the chemical mention is far away from the disease mention, especially they are not in one sentence. For example, a candidate < “AK”, “cisplatin” > with the context of *“The primary outcome was acute kidney injury (<ENTD> AKI <ENTD>). RESULTS: We evaluated 143 patients who received single-agent <ENTC> cisplatin <ENTC>”*, where *S*_1_is much longer and more complex than *S*_−1_ and *S*_0_, is wrongly labeled as 0 when without using the piecewise strategy, but correctly labeled as 1 when using the piecewise strategy. However, tackling the two types of cases above mentioned are still challenging. We evaluate the performance of our system (CNN + attention+piecewise in Table 3) on tackling cases when the chemical mention and disease mention are not in one sentence. The precision, recall, and F-score are only 53.15, 26.07 and 34.99% respectively.

Compared with CNN-based systems, our RPCNN-based system performs better. The main reason is that RPCNN provides a document-level representation for every candidate as all corresponding instances are considered, while CNN only selects one instance to represent a candidate by removing other instances where there may be different descriptions about relations.

There may be two limitations of our study: 1) chemical mentions and disease mentions themselves are ignored in the input layer. The chemcial and disease mentions may be helpful for CID extraction. In the future work, we will have a try to integrate chemical and disease mentions in the input layer for further improvement. 2) The effectiveness of our method is validated on an independent test set from the same resource (BioCreative V challenge), but not on latest papers. We will manually label a corpus from PubMed including latest papers as another separate test set for further validation.

## Conclusion

In this paper, we propose a novel document-level deep learning method for CID extraction. The proposed method naturally combines domain knowledge, piecewise strategy, attention mechanism and multi-instance learning together. The effectiveness of the method is validated on a benchmark corpus, and the system based on the proposed method shows competitive performance with other state-of-the-art systems.

## Abbreviations

CDR: Chemical-induced Disease Relation
CID: Chemical-induced Disease
CNN: Convolutional Neural Network
LSTM: Long Short Term Memory Neural Networks
RNN: Recurrent Neural Network
